# Clearing Extracellular Alpha-Synuclein from Cerebrospinal Fluid: A New Therapeutic Strategy in Parkinson’s Disease

**DOI:** 10.3390/brainsci8040052

**Published:** 2018-03-23

**Authors:** Manuel Menéndez-González, Huber S. Padilla-Zambrano, Cristina Tomás-Zapico, Benjamin Fernández García

**Affiliations:** 1 Servicio de Neurología, Hospital Universitario Central de Asturias, Oviedo 33011, Spain; 2 Department of Morphology and Cell Biology, University of Oviedo, Oviedo 33006, Spain; fernandezbenjamin@uniovi.es; 3 Instituto de Investigación Sanitaria del Principado de Asturias, Oviedo 33006, Spain; tomascristina@uniovi.es; 4 Estudiante de Medicina. Centro de Investigaciones Biomédicas (CIB), Faculty of medicine, University of Cartagena, Cartagena 130001, Colombia; huber_padilla20@hotmail.com; 5 Department of Functional Biology, University of Oviedo, Oviedo 33006, Spain

**Keywords:** Parkinson’s disease, alpha-synuclein, cerebrospinal fluid, immunotherapy, “CSF sink” hypothesis

## Abstract

This concept article aims to show the rationale of targeting extracellular α-Synuclein (α-Syn) from cerebrospinal fluid (CSF) as a new strategy to remove this protein from the brain in Parkinson’s disease (PD). Misfolding and intracellular aggregation of α-synuclein into Lewy bodies are thought to be crucial in the pathogenesis of PD. Recent research has shown that small amounts of monomeric and oligomeric α-synuclein are released from neuronal cells by exocytosis and that this extracellular alpha-synuclein contributes to neurodegeneration, progressive spreading of alpha-synuclein pathology, and neuroinflammation. In PD, extracellular oligomeric-α-synuclein moves in constant equilibrium between the interstitial fluid (ISF) and the CSF. Thus, we expect that continuous depletion of oligomeric-α-synuclein in the CSF will produce a steady clearance of the protein in the ISF, preventing transmission and deposition in the brain.

## 1. Extracellular α-Synuclein as a Target in Parkinson’s Disease

**Figure 1:**
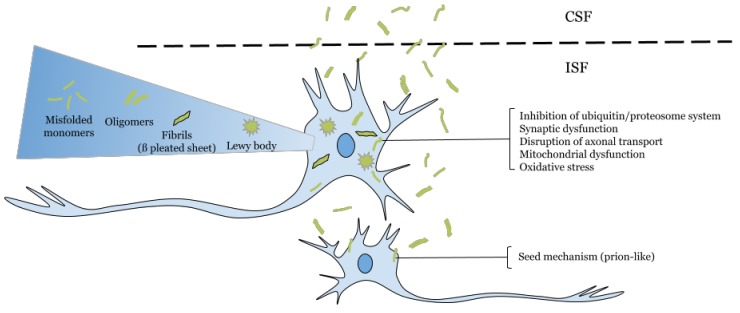
Effects of intracellular and extracellular alpha-synuclein. [10].

α-Synuclein (α-Syn) is a small protein comprising 140 amino acids with three domains: the N-terminally segment, a central hydrophobic region—also called the “non-amyloid component” or “NAC”—, and the C-terminal region, with an important role in the aggregation of the protein [1,2,3]. Under physiological conditions, α-Syn is in its native conformation as a soluble monomer [4]. Although the functions of α-Syn have not been elucidated, it has been associated with the suppression of apoptosis, the regulation of glucose levels, the modulation of calmodulin activity, playing a role as a molecular chaperone, the maintenance of levels of polyunsaturated fatty acids and antioxidants, neuronal differentiation, and the regulation of dopamine biosynthesis [5] and plays a role in maintaining a supply of synaptic vesicles in mature presynaptic terminals [6]. However, an influence of genetic mutations has been found in the N-terminal domain, speeding up aggregation, and the importance of the NAC domain in the formation of toxic α-Syn oligomers and fibrils [3], which could affect many cellular pathways and functions such as endocytosis, transport of ER to Golgi, the ubiquitin–proteasome system (UPS), autophagy, ER, and oxidative and nitration stress [7,8,9]. In α-synucleinopathies, an important characteristic is the presence of intracellular protein bodies containing α-Syn aggregates, known as Lewy bodies [6,10] (Figure 1).

Two hypotheses about the structure of the protein have been proposed: An alpha-helical folded tetramer and a tetramer structure that is only adopted after membrane binding [11,12,13,14]. Bartels et al. observed the status of endogenous α-Syn in human red blood cells, showing that this endogenous cellular protein exists natively as a 58 kDa helically folded tetramer [13]. In a study conducted in mice, the native form of the protein could be an unstructured monomer, which exhibits a random spiral structure [15]. 

The phosphorylation of α-Syn is essential in the degradation process [16,17]. The UPS degrades the soluble monomer of α-Syn and the autophagy-lysosomal pathway is responsible for the degradation of the most complex conformations [18,19]. The insoluble form of the protein has not been associated with neurotoxic effects although it is misfolded, but its monomeric and oligomeric forms present neurotoxic effects, being their propagation possible given by extracellular vesicles [20]. This suggests a toxicity not only to the central nervous system (CNS), but also to other systems, leading to an analysis of the relationship of the protein with non-motor symptoms (for example, the lack of olfactory sensation) of Parkinson’s disease (PD) [21,22]. This propagation becomes an important factor in the progression of PD [22,23,24,25,26,27,28,29]. It is believed that α-Syn is secreted, because it can be detected in human plasma and cerebrospinal fluid (CSF) [30,31]. Despite this mechanism is not yet known, it has been indicated its released by exosomes in a calcium-dependent manner [32]. Although the physiological structure and normal function of the protein are not fully understood, it is believed to (1) involve functions in the compartmentalization, storage, and recycling of neurotransmitters and the physiological regulation of certain enzymes, and (2) increase the number of dopamine transporter peptides molecules [33,34]. 

Recent research showed that small amounts of monomeric and oligomeric α-Syn are released from neuronal cells by unconventional exocytosis, and this extracellular α-Syn contributes to neurodegeneration, progressive spreading of α-Syn pathology, and neuroinflammation. Extracellular α-Syn is taken up by neurons through endocytosis and undergoes endocytic trafficking for degradation in lysosomes. Thus, α-Syn aggregates can be transmitted from neuron to neuron via the extracellular milieu and can propagate aggregates by a “seeding” mechanism. Moreover, extracellular α-Syn oligomers induce microglia via activation of Toll-like receptor 2 [35].

Several studies have shown a reduction in CSF α-Syn levels in PD [36,37,38] so it has been considered as a potential biomarker for the diagnosis of PD, but its sensitivity and specificity indicate that it will not be sufficient [39]. Plasmatic and urine vesicles derived from the CNS containing α-Syn might also be used as biomarkers in the diagnosis of PD [40].

To control the toxicity produced by α-Syn, some methods have been found: reducing α-Syn cleavage by caspase-1 [41], inducing protein clearance through neuronal autophagy [42], and cellular clearance through innate and adaptive immunization due to the proteotoxic mechanisms and inflammation that the protein induces [33]. In addition, antibodies directed to the sites of C-terminal truncation, oxidation, and nitration of alpha-synuclein might reduce the propagation and inhibit oligomerization, which would be a therapeutic potential [43].

## 2. Targeting Extracellular α-Synuclein at the Cerebrospinal Fluid

The immunological selection of oligomeric extracellular α-Syn in animal models accelerates the clearance of this protein [43]. The use of anti-α-Syn antibodies in transgenic mice promoted extracellular clearance mediated by microglia, preventing its transmission from cell to cell, as well as reducing neurodegeneration and functional deficits associated with its overexpression [43]. However, there might be a more efficient way of removing oligomeric α-Syn from the brain: removing it from the CSF. We propose that a therapeutic approach ensuring continuous flow from the brain interstitial fluid (ISF) to the CSF would remove extracellular α-Syn more effectively.

We have previously posed a new therapeutic hypothesis: that soluble peptides can be cleared from the brain with interventions where peptides are continuously removed from the CSF [21]. In other words, altering the levels of soluble proteins in the CSF also alters their levels in the brain parenchyma. In PD, extracellular oligomeric- α-Syn moves in constant equilibrium between the ISF and the CSF [20]. Thus, we expect continuous depletion of oligomeric- α-Syn in the CSF would produce a steady clearance of oligomeric- α-Syn in the ISF, preventing transmission and deposition in the brain. 

**Figure 2:**
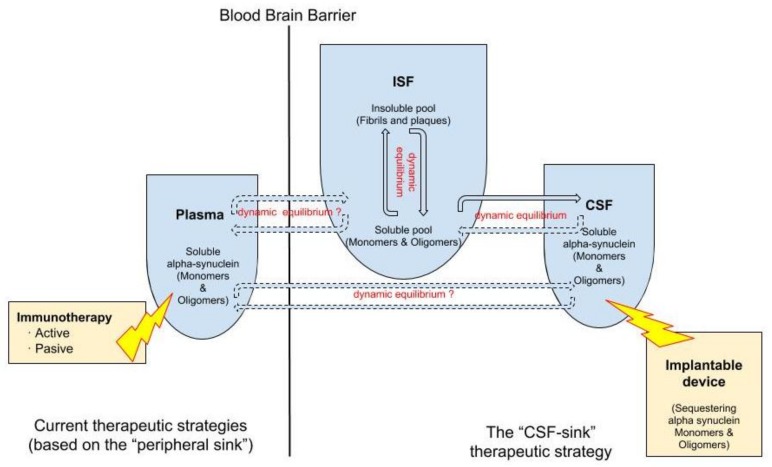
Double dynamic equilibrium of soluble α-Syn: there is a bidirectional equilibrium between insoluble and soluble pools of soluble α-Syn in the interstitial fluid (ISF) and there is a second equilibrium, also probably bidirectional, of soluble α-Syn between the ISF and the cerebrospinal fluid (CSF). The “CSF sink therapeutic strategy” consists in sequestering target proteins from the CSF with implantable devices, thus inducing changes in the levels of these proteins in the ISF.

Today, we can conceive several innovative ways of removing peptides continuously from the CNS by accessing the CSF and debugging it using filtration devices. For instance, peptides can be targeted either by size—particularly the aggregated forms—or by immunological techniques, or by a combination of both. These devices can be endowed with different technologies able to capture target molecules, such as oligomeric α-Syn , from the CSF. Thus, these interventions would work as a central sink of α-Syn, reducing the levels of CSF oligomeric α-Syn, and, by means of the CSF-ISF equilibrium, would promote the efflux of oligomeric α-Syn from the ISF to the CSF (Figure 2).

## 3. Conclusions

We introduce here a rationale for the “CSF sink” hypothesis and conclude that continuous depletion of oligomeric α-Syn in the CSF will produce a steady clearance of oligomeric α-Syn in the ISF. Implantable devices aimed at sequestering oligomeric α-Syn from the CSF may represent a new therapeutic strategy in PD and other α-synucleinopathies.
